# Supplementary material for: Incidence of Lower Extremity Amputation in Romania: A Nationwide 5-Year Cohort Study, 2015–2019
Source: Medicina (Kaunas). 2023 Jun 25;59(7):1199. doi: 10.3390/medicina59071199 (PMC10385247; doi:10.3390/medicina59071199)

Supplementary Table 1. Number of DR-LEAs distributed by sex, age groups, type of amputation

Diabetes-related LEAs	*Sex*			*Age groups (years)*					
	**Male**	**Female**	**Total**	**<30**	**30-39**	**40-49**	**50-59**	**60-69**	**>=70**
2015 DR-LEAs	**5173**	**2011**	**7184**	**6**	**45**	**360**	**1331**	**2856**	**2586**
2015 Major DR-LEAs	*1416*	*744*	*2160*	*1*	*5*	*78*	*326*	*810*	*940*
2015 Minor DR-LEAs	*3757*	*1267*	*5024*	*5*	*40*	*282*	*1005*	*2046*	*1646*
2016 DR-LEAs	**5496**	**2109**	**7605**	**5**	**34**	**374**	**1432**	**3007**	**2753**
2016 Major DR-LEAs	*1501*	*730*	*2231*	*0*	*6*	*65*	*338*	*837*	*985*
2016 Minor DR-LEAs	*3995*	*1379*	*5374*	*5*	*28*	*309*	*1094*	*2170*	*1768*
2017 DR-LEAs	**5958**	**2276**	**8234**	**9**	**36**	**402**	**1407**	**3346**	**3034**
2017 Major DR-LEAs	*1624*	*745*	*2369*	*0*	*6*	*81*	*303*	*908*	*1071*
2017 Minor DR-LEAs	*4334*	*1531*	*5865*	*9*	*30*	*321*	*1104*	*2438*	*1963*
2018 DR-LEAs	**6159**	**2360**	**8519**	**6**	**43**	**370**	**1480**	**3446**	**3174**
2018 Major DR-LEAs	*1664*	*799*	*2463*	*1*	*9*	*81*	*306*	*967*	*1099*
2018 Minor DR-LEAs	*4495*	*1561*	*6056*	*5*	*34*	*289*	*1174*	*2479*	*2075*
2019 DR LEAs	**6547**	**2410**	**8957**	**3**	**36**	**371**	**1470**	**3532**	**3545**
2019 Major DR-LEAs	*1701*	*768*	*2469*	*0*	*6*	*77*	*313*	*925*	*1148*
2019 Minor DR-LEAs	*4846*	*1642*	*6488*	*3*	*30*	*294*	*1157*	*2607*	*2397*
Total DR-LEAs (2015-2019)	**29333**	**11166**	**40499**	**29**	**194**	**1877**	**7120**	**16187**	**15092**
Major DR-LEAs (2015-2019)	*7906*	*3786*	*11692*	*2*	*32*	*382*	*1586*	*4447*	*5243*
Minor DR-LEAs (2015-2019)	*21427*	*7380*	*28807*	*27*	*162*	*1495*	*5534*	*11740*	*9849*

Supplementary Table 2. Number of ATSR-LEAs divided by sex, age groups, type of amputation

ATS related LEAs	*Sex*			*Age groups (years)*					
	**Male**	**Female**	**Total**	**<30**	**30-39**	**40-49**	**50-59**	**60-69**	**>=70**
2015 Total ATSR LEAs	**5409**	**1718**	**7127**	5	55	273	1099	2292	3403
2015 ATSR-Major A 2015	*2584*	*899*	*3483*	*3*	*21*	*104*	*479*	*1084*	*1792*
2015 ATSR-minor A 2015	*2825*	*819*	*3644*	*2*	*34*	*169*	*620*	*1208*	*1611*
2016 Total ATSR LEAs	**5708**	**1928**	**7636**	4	43	295	1110	2604	3580
2016 Major LEAs ATSR	*2638*	*955*	*3593*	*2*	*14*	*94*	*479*	*1168*	*1836*
2016 Minor LEAs ATSR	*3070*	*973*	*4043*	*2*	*29*	*201*	*631*	*1436*	*1744*
2017 Total ATSR LEAs	**5887**	**1843**	**7730**	5	50	291	994	2740	3650
2017 Major LEAs ATSR	*2655*	*891*	*3546*	*0*	*10*	*107*	*365*	*1219*	*1845*
2017 Minor LEAs ATSR	*3232*	*952*	*4184*	*5*	*40*	*184*	*629*	*1521*	*1805*
2018 Total ATSR LEAs	**6043**	**1945**	**7988**	4	40	294	1003	2776	3871
2018 Major LEAs ATSR	*2783*	*955*	*3738*	*2*	*11*	*96*	*408*	*1222*	*1999*
2018 Minor LEAs ATSR	*3260*	*990*	*4250*	*2*	*29*	*198*	*595*	*1554*	*1872*
2019 Total ATSR LEAs	**6019**	**2090**	**8109**	7	33	266	1018	2718	4067
2019 Major LEAs ATSR	*2667*	*982*	*3649*	*0*	*7*	*100*	*375*	*1185*	*1982*
2019 Minor LEAs ATSR	*3352*	*1108*	*4460*	*7*	*26*	*166*	*643*	*1533*	*2085*
Total ATSR LEAs (2015-2019)	**29066**	**9524**	**38590**	25	221	1419	5224	13130	18571
Major LEAs ATSR (2015-2019)	*13327*	*4682*	*18009*	*7*	*63*	*501*	*2106*	*5878*	*9454*
Minor LEAs ATSR (2015-2019)	*15739*	*4842*	*20581*	*18*	*158*	*918*	*3118*	*7252*	*9117*

Supplementary Table 3. Number of Trauma-LEAs divided by sex, age groups, type of amputation

Trauma related LEAs	*Sex*			*Age groups (years)*					
	**Male**	**Female**	**Total**	**<30**	**30-39**	**40-49**	**50-59**	**60-69**	**>=70**
2015 TraumaR-LEAs	1354	357	1711	82	58	172	356	562	481

2015 Major TraumaR-LEAs	652	154	806	32	25	62	169	261	257
2015 Minor TraumaR-LEAs	702	203	905	50	33	110	187	301	224
2016 TraumaR-LEAs	1326	376	1702	60	55	173	339	538	537
2016 Major TraumaR-LEAs	679	186	865	20	34	71	168	262	310
2016 Minor TraumaR-LEAs	647	190	837	40	21	102	171	276	227
2017 TraumaR-LEAs	1323	394	1717	62	52	149	354	562	538
2017 Major TraumaR-LEAs	658	197	855	20	23	70	160	269	313
2017 Minor TraumaR-LEAs	665	197	862	42	29	79	194	293	225
2018 TraumaR-LEAs	1467	416	1883	58	66	198	372	617	572
2018 Major TraumaR-LEAs	700	208	908	28	26	80	160	295	319
2018 Minor TraumaR-LEAs	767	208	975	30	40	118	212	322	253
2019 TraumaR LEAs	1589	411	2000	56	54	174	400	703	613
2019 Major TraumaR-LEAs	758	180	938	25	21	90	167	323	312
2019 Minor TraumaR-LEAs	831	231	1062	31	33	84	233	380	301
Total TraumaR-LEAs (2015-2019)	7059	1954	9013	318	285	866	1821	2982	2741
Major TraumaR-LEAs (2015-2019)	3447	925	4372	125	129	373	824	1410	1511
Minor TraumaR-LEAs (2015-2019)	3612	1029	4641	193	156	493	997	1572	1230

Supplementary Table 4. Incidence of LEAs by type, sex and age group

Incidence	Sex			Age groups (years)					
	Male	Female	Total	<30	30-39	40-49	50-59	60-69	>=70
**2015 DR-LEAs**	26,03	10,12	36,14	0,03	0,23	1,81	6,70	14,37	13,01
**2015 Major DR-LEAs**	7,12	3,74	10,87	0,01	0,03	0,39	1,64	4,08	4,73
**2015 Minor DR-LEAs**	18,90	6,37	25,28	0,03	0,20	1,42	5,06	10,29	8,28
**2016 DR-LEAs**	27,81	10,67	38,49	0,03	0,17	1,89	7,25	15,22	13,93
**2016 Major DR-LEAs**	7,60	3,69	11,29	0,00	0,03	0,33	1,71	4,24	4,98
**2016 Minor DR-LEAs**	20,22	6,98	27,20	0,03	0,14	1,56	5,54	10,98	8,95
**2017 DR-LEAs**	30,33	11,59	41,92	0,05	0,18	2,05	7,16	17,03	15,44
**2017 Major DR-LEAs**	8,27	3,79	12,06	0,00	0,03	0,41	1,54	4,62	5,45
**2017 Minor DR-LEAs**	22,06	7,79	29,86	0,05	0,15	1,63	5,62	12,41	9,99
**2018 DR-LEAs**	31,53	12,08	43,61	0,03	0,22	1,89	7,58	17,64	16,25
**2018 Major DR-LEAs**	8,52	4,09	12,61	0,01	0,05	0,41	1,57	4,95	5,63
**2018 Minor DR-LEAs**	23,01	7,99	31,00	0,03	0,17	1,48	6,01	12,69	10,62
**2019 DR LEAs**	33,70	12,41	46,11	0,02	0,19	1,91	7,57	18,18	18,25
**2019 Major DR-LEAs**	8,76	3,95	12,71	0,00	0,03	0,40	1,61	4,76	5,91
**2019 Minor DR-LEAs**	24,95	8,45	33,40	0,02	0,15	1,51	5,96	13,42	12,34
**Total DR-LEAs (2015-2019)**	29,86	11,37	41,22	0,03	0,20	1,91	7,25	16,48	15,36
**Major DR-LEAs (2015-2019)**	8,05	3,85	11,90	0,00	0,03	0,39	1,61	4,53	5,34
**Minor DR-LEAs (2015-2019)**	21,81	7,51	29,32	0,03	0,16	1,52	5,63	11,95	10,03
**2015 Total ATSR LEAs**	27,21	8,64	35,86	0,03	0,28	1,37	5,53	11,53	17,12
**2015 ATSR-Major A 2015**	13,00	4,52	17,52	0,02	0,11	0,52	2,41	5,45	9,02
**2015 ATSR-minor A 2015**	14,21	4,12	18,33	0,01	0,17	0,85	3,12	6,08	8,11
**2016 Total ATSR LEAs**	28,89	9,76	38,64	0,02	0,22	1,49	5,62	13,18	18,12
**2016 Major LEAs ATSR**	13,35	4,83	18,18	0,01	0,07	0,48	2,42	5,91	9,29
**2016 Minor LEAs ATSR**	15,54	4,92	20,46	0,01	0,15	1,02	3,19	7,27	8,83
**2017 Total ATSR LEAs**	29,97	9,38	39,35	0,03	0,25	1,48	5,06	13,95	18,58
**2017 Major LEAs ATSR**	13,52	4,54	18,05	0,00	0,05	0,54	1,86	6,21	9,39
**2017 Minor LEAs ATSR**	16,45	4,85	21,30	0,03	0,20	0,94	3,20	7,74	9,19
**2018 Total ATSR LEAs**	30,94	9,96	40,89	0,02	0,20	1,51	5,13	14,21	19,82
**2018 Major LEAs ATSR**	14,25	4,89	19,14	0,01	0,06	0,49	2,09	6,26	10,23
**2018 Minor LEAs ATSR**	16,69	5,07	21,76	0,01	0,15	1,01	3,05	7,96	9,58
**2019 Total ATSR LEAs**	30,98	10,76	41,74	0,04	0,17	1,37	5,24	13,99	20,94

**2019 Major LEAs ATSR**	13,73	5,06	18,78	0,00	0,04	0,51	1,93	6,10	10,20
**2019 Minor LEAs ATSR**	17,26	5,70	22,96	0,04	0,13	0,85	3,31	7,89	10,73
**Total ATSR LEAs (2015-2019)**	29,59	9,69	39,28	0,03	0,22	1,44	5,32	13,37	18,90
**Major LEAs ATSR (2015-2019)**	13,57	4,77	18,34	0,01	0,06	0,51	2,14	5,98	9,62
**Minor LEAs ATSR (2015-2019)**	16,02	4,93	20,95	0,02	0,16	0,93	3,17	7,38	9,28
**2015 TraumaR-LEAs**	6,81	1,80	8,61	0,41	0,29	0,87	1,79	2,83	2,42
**2015 Major TraumaR-LEAs**	3,28	0,77	4,06	0,16	0,13	0,31	0,85	1,31	1,29
**2015 Minor TraumaR-LEAs**	3,53	1,02	4,55	0,25	0,17	0,55	0,94	1,51	1,13
**2016 TraumaR-LEAs**	6,71	1,90	8,61	0,30	0,28	0,88	1,72	2,72	2,72
**2016 Major TraumaR-LEAs**	3,44	0,94	4,38	0,10	0,17	0,36	0,85	1,33	1,57
**2016 Minor TraumaR-LEAs**	3,27	0,96	4,24	0,20	0,11	0,52	0,87	1,40	1,15
**2017 TraumaR-LEAs**	6,73	2,01	8,74	0,32	0,26	0,76	1,80	2,86	2,74
**2017 Major TraumaR-LEAs**	3,35	1,00	4,35	0,10	0,12	0,36	0,81	1,37	1,59
**2017 Minor TraumaR-LEAs**	3,39	1,00	4,39	0,21	0,15	0,40	0,99	1,49	1,15
**2018 TraumaR-LEAs**	7,51	2,13	9,64	0,30	0,34	1,01	1,90	3,16	2,93
**2018 Major TraumaR-LEAs**	3,58	1,06	4,65	0,14	0,13	0,41	0,82	1,51	1,63
**2018 Minor TraumaR-LEAs**	3,93	1,06	4,99	0,15	0,20	0,60	1,09	1,65	1,30
**2019 TraumaR LEAs**	8,18	2,12	10,30	0,29	0,28	0,90	2,06	3,62	3,16
**2019 Major TraumaR-LEAs**	3,90	0,93	4,83	0,13	0,11	0,46	0,86	1,66	1,61
**2019 Minor TraumaR-LEAs**	4,28	1,19	5,47	0,16	0,17	0,43	1,20	1,96	1,55
**Total TraumaR-LEAs (2015-2019)**	7,19	1,99	9,17	0,32	0,29	0,88	1,85	3,04	2,79
**Major TraumaR-LEAs (2015-2019)**	3,51	0,94	4,45	0,13	0,13	0,38	0,84	1,44	1,54
**Minor TraumaR-LEAs (2015-2019)**	3,68	1,05	4,72	0,20	0,16	0,50	1,01	1,60	1,25

Supplementary Table 5. Incidence of major DR-LEAs by sex

Year	Incidence of major amputations in men with diabetes	Incidence of major amputations in women with diabetes	Total incidence of major amputations in patients with diabetes
2015	14.58	7.32	10.87
2016	15.55	7.22	11.29
2017	16.91	7.42	12.06
2018	17.42	8.01	12.61
2019	17.89	7.74	12.71
Total	16.47	7.54	11.91

Supplementary Table 6. Incidence of minor DR-LEAs distributed by sex

Year	Incidence of minor amputations in men with diabetes	Incidence of minor amputations in women with diabetes	Total incidence of minor amputations in patients with diabetes
2015	38.69	12.46	25.28
2016	41.40	13.64	27.20
2017	45.14	15.25	29.86
2018	47.04	15.64	31.00
2019	50.98	16.55	33.40
Total	44.65	14.71	29.35

Supplementary Figure 1,2. Incidence of LEAs under and over 60 years of age

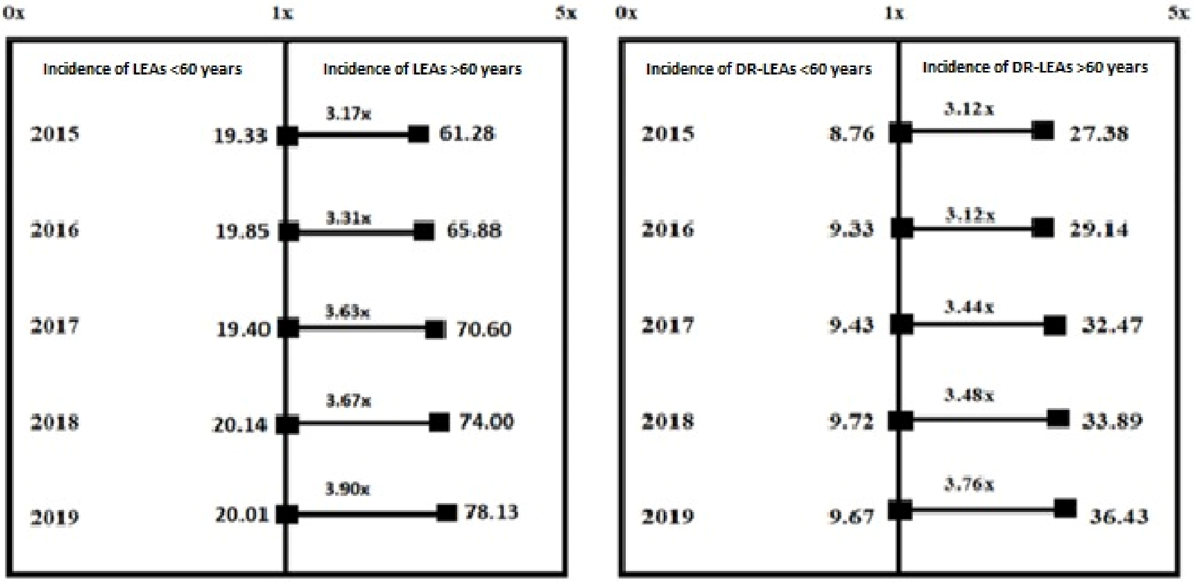

Supplement: Supplementary file 1 [file medicina-59-01199-s001.zip › medicina-2432429-supplementary.pdf]
